# Study on Two Typical Progressive Motions in Tai Chi (Bafa Wubu) Promoting Lower Extremity Exercise

**DOI:** 10.3390/ijerph20032264

**Published:** 2023-01-27

**Authors:** Haojie Li, Fang Peng, Shaojun Lyu, Zhongqiu Ji, Yameng Li

**Affiliations:** 1 School of Physical Education and Sports, Beijing Normal University, Beijing 100875, China; 2 Department of Physical Education, Peking University, Beijing 100871, China

**Keywords:** Tai Chi Chuan, sport, finite element, musculoskeletal modeling, lower limbs

## Abstract

Background: By comparatively investigating the joints, muscles and bones of the lower extremity during two progressive motions in Bafa Wubu and normal walking, this paper aims to enrich the diversity of walking exercise and scientifically provide theoretical guidance for primary practitioners. The scientific training methods and technical characteristics of Bafa Wubu, as well as its contribution to comprehensive exercise of the lower extremities, are further explored. Methods: A total of eight professional athletes of Tai Chi at the national level were recruited. The kinetic parameters of the lower extremity were calculated using AnyBody 7.2 musculoskeletal modeling. Stress analysis of the iliac bone was performed using an ANSYS 19.2 workbench. Results: In Bafa Wubu, the ground reaction force during two progressive motions was significantly smaller than that noted during normal walking. During warding off with steps forward and laying with steps forward, the load at the three joints of the lower extremity was significantly smaller than that during normal walking in the frontal plane, but significantly greater than that noted during normal walking in the vertical axis. In addition, the lower limb joint torque was higher than that of normal walking in both progressive movements, and lower limb muscle activation was higher. The iliac bone loads during the two progressive motions were larger than those during normal walking, and the maximum loading point differed. Conclusions: This is the first study to demonstrate the biomechanical performance of Bafa Wubu in professional athletes of Tai Chi. Two progressive motions of Bafa Wubu require the lower extremity to be slowly controlled, thereby resulting in a smaller ground reaction force. In addition, the loads of the three joints at the lower extremity all increase in the vertical direction and decrease in the lateral direction, reducing the possibility of lateral injury to the joints. In addition, the two progressive motions significantly enhance the muscle strength of the plantar flexion muscles, dorsiflexor, and muscles around the thigh, and effectively stimulate the bones of the lower extremity. Therefore, progressive motion training contributes to improving the controlling and supporting capabilities of the lower extremities during normal walking.

## 1. Introduction

As a slow and gentle moderate-intensity aerobic exercise, Tai Chi Chuan (TC) has been widely used as a therapeutic intervention in rehabilitation as well as in fitness for more than 10 years worldwide [1]. Past studies have shown that TC exercise can strengthen the lower extremity [2], reduce bone density loss [3], improve balance [4], and prevent falls [5].

Tai Chi (Bafa Wubu), which is studied in this paper, is a new type of simplified Tai Chi that is suitable for practice by the general population, such as older people whose cognitive ability and ability to perform and learn decreases with age [6,7]. Older people have difficulty completing complex tasks [8]; as traditional Tai Chi training methods are more complex for older people, it is difficult to practice [9]. Considering that the traditional form of TC is difficult for elderly individuals, simplified and personalized versions of TC have been developed to facilitate learning of TC [10]. Hence, the General Administration of Sport of China promotes a more user-friendly routine of TC, based on the 24-form simplified TC, called Bafa Wubu. It integrates eight essential forms called Peng (warding off), Lv (rolling back), Ji (pressing), An (pushing), Cai (plucking), Lie (laying), Zhou (elbowing), Kao (leaning sideways), and five steps called Jin (advancing), Tui (retreating), Gu (shifting left), Pan (shifting right), and Ding (central equilibrium), which are the core patterns of Tai Chi. Compared with the 24-form simplified version, Bafa Wubu is even simpler and takes less time and energy for beginners of TC [11]. Therefore, it is of great value to probe into Bafa Wubu’s role and mechanism of promoting fitness. Tsang [12] found that by practicing Tai Chi, elderly individuals could enhance their proprioception, promote their vestibular function, and strengthen their lower extremities, subsequently improving their dynamic and static balance. Likewise, some scholars believe that Bafa Wubu improves the foot center of pressure (COP) and foot arch tactile sense more than knee proprioception, enhancing balance capacity and reducing the risk of falls in the elderly [13]. Additionally, some studies have focused on the typical actions of Bafa Wubu and reported its characteristics; for example, the motions have bilateral symmetry, proximal muscles work before the distal ones, and the lower extremity muscles play a major role in performing Bafa Wubu [14]. In addition, in Bafa Wubu, footwork basically moves forward, backward, left, right and center, and these different directions of footwork movement have positive implications for lower limb exercise [15]. According to existing literature, the Bafa Wubu form of Tai Chi was launched relatively recently, and biomechanical research on the topic is limited. There are no existing studies investigating the biomechanical performance of Bafa Wubu. In this study, the AnyBody Musculoskeletal Model was utilized to analyze the biomechanical characteristics of the lower extremities during two progressive motions of Bafa Wubu. In addition, changes in iliac bone load during the two progressive motions and normal walking were investigated using AnyBody finite element analysis. The purpose of this study was to investigate the biomechanical performance of two progressive motions of Bafa Wubu in strengthening the joints, muscles and bones of the lower extremity, compared with walking exercise. Our findings may provide scientific evidence of TC exercise improving muscle strength and reducing joint compression force.

Research hypothesis: (1) In Bafa Wubu’s two progressive movements, the ground reaction force is less and the control of lower limbs is required. (2) The joint forces of the two progressive movements of Bafa Wubu are higher than those of normal walking. (3) Muscle activation is different for different movements. (4) The two progressive movements of Bafa Wubu exercise the iliac bone stress force to a higher degree than normal walking.

## 2. Materials and Methods

### 2.1. Participants

A total of 8 male TC athletes from Beijing Sport University were recruited as subjects. Only professional athletes who had achieved both national level titles or top three athletes in domestic TC competitions were qualified. The athletes were expected to be in good health, without injury to their lower extremity in the past six months. After screening for eligibility for this study, a selected total of 5 national master sportsmen and 3 national athletes were recruited. All participants provided written informed consent and understood the experimental process and purpose. This study was reviewed and approved by the Ethics Committee of the China National Rehabilitation Center. The participants’ basic information is listed in Table 1.

### 2.2. Methods

#### 2.2.1. Instrument

All motion data were collected using a high-precision motion capture system with 8 infrared cameras (BTS SMART DX 700, BTS Bioengineering, Italy, Rome) based on the following parameters: frequency of 250 Hz, resolution of 640 × 480 pixels, and precision of 400 mm × 300 mm × 300 mm. Three 3D force plates (928E, Kistler, Switzerland, Bern) were used to measure the ground reaction force with a sampling frequency of 1000 Hz.

#### 2.2.2. Two Progressive Motions in Tai Chi (Bafa Wubu)

**Figure 1:**
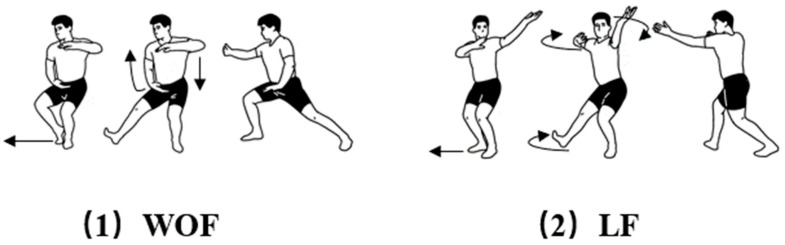
Illustration of the Two Progressive Motions of Bafa Wubu of Tai Chi: (**1**) WOF, warding off with steps forward; (**2**) LF, laying with steps forward.

Bafa Wubu, a primary routine of TC, is composed of five types of footwork called Jin (advancing), Tui (retreating), Gu (shifting left), Pan (shifting right), and Ding (central equilibrium), and eight essential forms called Peng (warding off), Lv (rolling back), Ji (pressing), An (pushing), Cai (plucking), Lie (laying), Zhou (elbowing), and Kao (leaning sideways). As shown in Figure 1, warding off with steps forward (WOF) and laying with steps forward (LF) are two progressive motions of Bafa Wubu. WOF and LF represent forward movements, sharing similar motion features with NW (normal walking). However, WOF involves force outwards, upward, and forward from inside, and LF involves force outwards, forward and lateral rotation from inside. They also differ in strength generating modes and exercise effects. Therefore, this study mainly focused on the differences between NW and two progressive motions of Bafa Wubu.

#### 2.2.3. AnyBody Modeling

A musculoskeletal simulation model was established using AnyBody 7.2 software (AnyBody Technology, Denmark, Copenhagen) to process 3D motion dynamics. AnyBody has been validated by many studies and is recognised for high reliability and precision [16]. The musculoskeletal model established in this study is a standard multibody dynamic model, which consists of rigid parts (such as the human skeleton or external objects), kinematic actuators (such as body motion), and force/torque actuators (such as muscles). Generally, forces and torques are simulated by means of multibody dynamics simulations.

### 2.3. Testing Protocols

This experiment was conducted in the sports biomechanics laboratory of Beijing Normal University. We set the BTS infrared motion capture cameras at intervals of greater than 200 mm and at a height of 300 mm. The cameras were placed in a semicircle around the test center. Before the experiment, the global coordinates and force plate coordinates were calibrated to ensure that each camera could capture the participants’ motions and body segments. We turned on the surface EMG system and connected it to the infrared action system to synchronize motion and electromyographic signals. A VIXTA recording camera was used to record the whole experiment.

**Figure 2:**
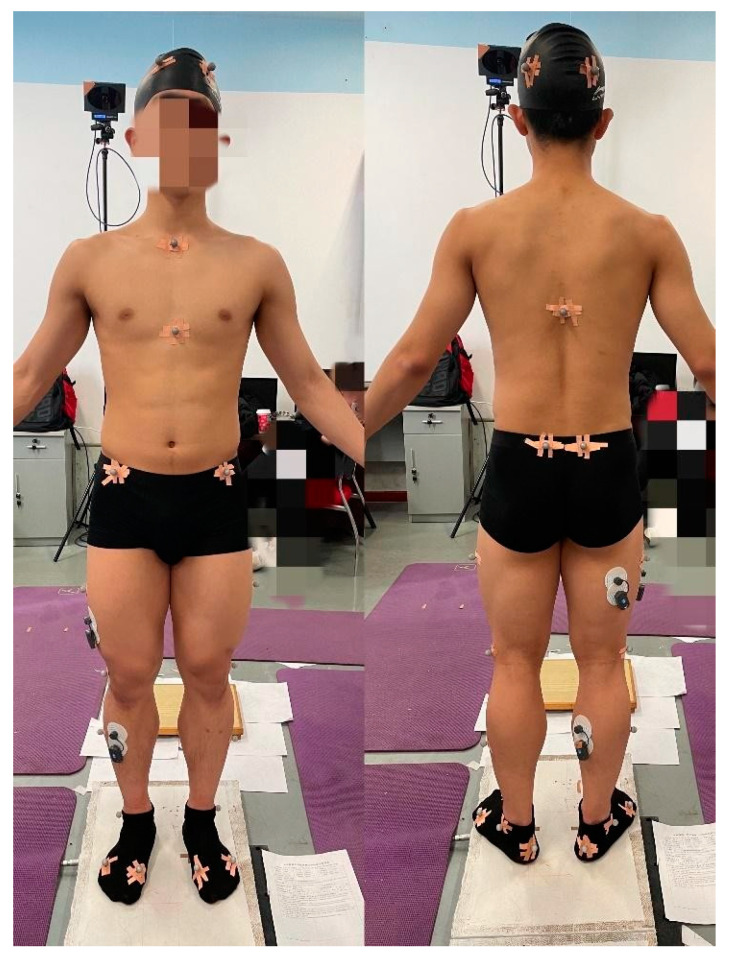
25 marker points were attached to the bone marker points of the subject.

Participants wore uniform shorts and black socks to reduce errors due to clothing shaking and other variables. According to the requirements of the Plug-in-Gait and the marker set of the lower extremity model in AnyBody 7.2, 25 marker points were attached to the bone marker points of the subject (Figure 2). The positions of these marker points included the left and right anterior head, left and right posterior head, sternum, clavicle joint, tenth thoracic vertebra, sternal xiphoid process, left and right anterior superior iliac spine, left and right posterior superior iliac spine, left and right lateral lower 1/3 of the thigh, left and right external epicondyle of the fibula, right lateral lower 1/3 of the calf, left lateral lower ½ of the leg, left and right heel, left and right lateral malleolus, left and right first metatarsal head, and left and right fifth metatarsal head [17].

### 2.4. Data Collection and Analysis

Given that all participants were right-side dominant and that the motions in the Bafa Wubu form of TC are symmetric and periodic, this experiment mainly investigated the characteristics of motions when the force of the knee joint in the vertical direction reached the maximum in the right lower extremity during the stance phase, which lasted from heel strike to toe off [18]. Starting from a standing position, participants performed normal walking and two progressive motions of Bafa Wubu on the force plate. Each motion was scheduled three times, and the average value of the three data peaks was recorded. These three groups of motions were finished in 4–6 s. The data selection range was the period: right foot lifting—right heel landing—right toe off the ground. 800 frames of images were selected for all three motions.

#### 2.4.1. Force Plate and Electromyography Data Processing

The force plate and electromyography data were processed using a BTS SMART Analyzer. We determined the range of the force plate according to the data and determined its maximum values from periods of force in the X, Y and Z axes [19]. After calculation, the data of the ground reaction force, lower extremity joint force, and muscle strength were divided by body weight as a normalization process. [20].

#### 2.4.2. Kinematic and Dynamic Data of the Lower Extremities

**Figure 3:**
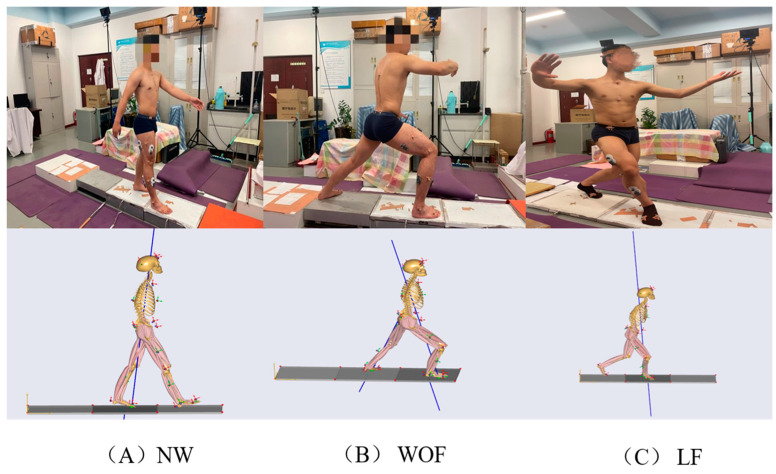
Musculoskeletal models of (**A**) NW, (**B**) WOF, (**C**) LF.

We used BTS SMART Capture software and eight BTS SMART Dx 700 cameras to capture the motion data of the reflective markers and then delineated their moving paths using the BTS tracker. When the process was completed, the C3D file of kinematic data was imported into AnyBody 7.2 simulation software to establish musculoskeletal simulation models of two progressive motions of Bafa Wubu and NW, respectively (Figure 3). The calculation process of the AnyBody simulation is performed as follows: optimizing the coordinates of reflective markers, conducting dynamics computation, and conducting inverse dynamics computation. Optimizing the coordinates of the reflective markers helps to enhance the accuracy of dynamics computation and make the model suit subjects as closely as possible so that inverse dynamics can be computed more accurately.

#### 2.4.3. Skeleton Finite Element Model Processing

The skeleton finite element model in this experiment was established based on a CT scans of two iliac bones of a healthy young man (171 cm, 75 kg). In Mimics 20.0 (Materialise, Leuven, Belgium), the original CT image was converted into tetrahedron elements and exported in STL format. Then, the bone and retrograde mesh were restored in Geomagic Wrap 2013 (Geomagic Company, Research Triangle Park, NC, USA), where the holes were filled and the sharp edges were repaired. The IGES format was exported after the treatment was completed. Finally, the iliac stress analysis was completed in ANSYS 19.2 workbench (SwansonAnalysis Inc., Houston, PA, USA). Compared with Toyohara’s study, this paper focused on the same iliac bone of young people as those in Toyohara’s study, which proved the effectiveness of bones. Hence, the present study uses the same Young’s modulus and Poisson’s ratio. The Young’s modulus (11,000 MPa) and Poisson’s ratio (0.2) of the iliac bone were added [21]. In addition, the finite element model was validated by fracture risk, consistent with Marco’s results, and it was confirmed that the skeletal model is reliable [22], where the sacroiliac joint surface and femoral head were fixed, and the net joint force was loaded to the end of the medial. Then, the force of the iliac bones was computed.

Mesh generation: The finite element model was meshed using a 1 mm 2nd order tetrahedral element which had a total number of elements and nodes of 126,324 and 61,545, respectively. The average mass of the elements was 0.80, indicating good mesh quality [23].

Loads and boundary constraints: This experiment was conducted in a static simulation environment. In order to correctly simulate the motion conditions of the progressive action in Bafa Wubu, the ANSYS FE analysis plug-in function in AnyBody software was used to export the joint forces and boundary conditions required by the finite element software [24]. The iliac bone model and joint forces were simultaneously imported into the workbench software for loading, and the sizes and positions of the muscle attachment points were manually adjusted to match the normal anatomical position and size of muscles. Taking into consideration the bottom-to-top force characteristics of Tai Chi, the simulation best matched the force characteristics of Tai Chi, and thus the hip joint force was selected for analysis, for loading and boundary constraints of the forces around the iliac bone.

#### 2.4.4. Statistical Analysis

Data were analyzed with statistical analysis software (SPSS 26.0), and the results are displayed as the mean value and standard deviation (mean ± SD). A paired samples t-test was used to analyze ground reaction force, joint force, and joint torque during two progressive motions of Bafa Wubu and NW. *p* < 0.05 represents a statistically significant difference.

## 3. Results

### 3.1. Comparisons of Ground Reaction Force

**Figure 4:**
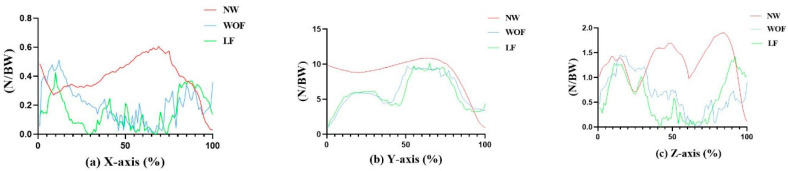
Comparisons of the GRF during NW (red line), WOF (blue line) and LF (green line): (**a**) GRF on the X-axis, (**b**) GRF on the Y-axis, and (**c**) GRF on the Z-axis.

As shown in Table 2 and Figure 4, during WOF, the ground reaction force (GRF) is significantly smaller than that noted during NW in the vertical and sagittal axes (*p* < 0.01). During LF, GRF is significantly smaller than that noted during NW on the sagittal axis (*p* < 0.01).

### 3.2. Comparisons of Joint Load of the Lower Extremity

The joint loads exerted at the ankle, knee and hip joints are shown, with peak values, in Table 3. During WOF and LF, the hip joint load was significantly smaller than that during NW on the frontal axis (*p* < 0.01), whereas it was significantly greater than that noted during NW on the vertical and sagittal axes (*p* < 0.01). The knee joint load during the two progressive motions was significantly smaller than that noted during NW on the frontal axis (*p* < 0.01) and significantly greater than that observed during NW on the vertical axis (*p* < 0.01). The ankle joint load during the two progressive motions was significantly smaller than that noted during NW on the frontal axis (*p* < 0.01) and greater than that observed during NW on the vertical axis (*p* < 0.05).

During WOF, knee flexion/extension torque and ankle flexion/extension torque were significantly larger than those observed during NW (*p* < 0.01). During LF, hip abduction torque was greater than that observed during NW (*p* < 0.05); hip flexion/extension torque and ankle flexion/extension torque were significantly greater than those observed during NW.

### 3.3. Comparisons of Muscle Strength of the Lower Extremities

The muscle strength values of the lower extremities during two progressive motions and NW are shown in Table 4. During WOF, the muscle strength values of the vastus lateralis, vastus medialis, tensor fasciae latae, gluteus maximus, iliopsoas, piriformis, adductor magnus, tibialis anterior, and gastrocnemius were significantly greater than those noted during NW (*p* < 0.01). During LF, the muscle strength values of the vastus lateralis, vastus medialis, tensor fasciae latae, gluteus maximus, gluteus minimus, iliopsoas, piriformis, adductor magnus, and gastrocnemius were significantly greater than those observed during NW (*p* < 0.01).

### 3.4. Comparisons of Iliac Bone Stress

**Figure 5:**
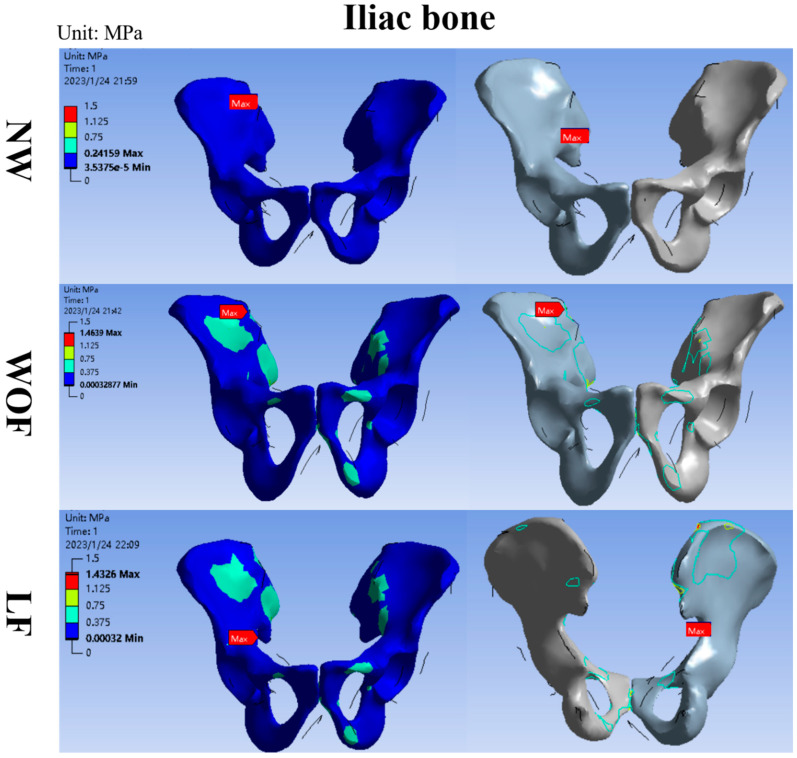
Iliac bone stress distribution cloud map. NW, normal walking; WOF, warding off with steps forward; LF, laying with steps forward. Equivalent stress distribution of the iliac bone during two progressive motions of Bafa Wubu and NW is shown. The warmer the color, the greater the stress. The red arrow indicates the maximum stress, and black lines around bones indicate their displacement distance.

**Figure 6:**
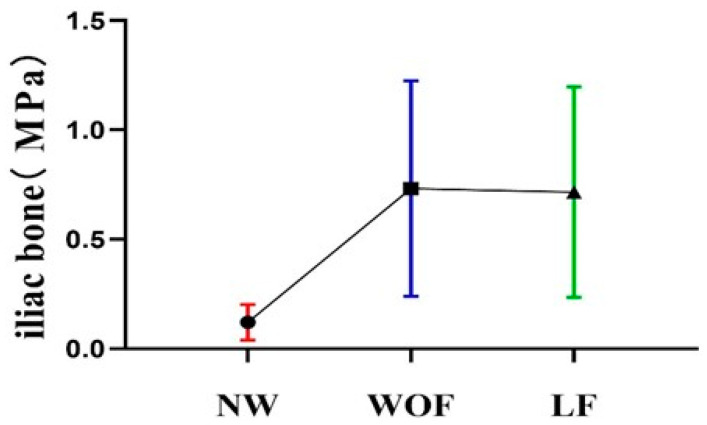
Mean values of stress distribution on the iliac bone during two progressive motions of Bafa Wubu and NW (NW in red; WOF in blue; LF in green).

The equivalent stress distribution of the skeleton during two progressive motions of Bafa Wubu and NW is depicted in Figure 5 and Figure 6. During WOF and LF, stress on the iliac bone is significantly greater than that noted during NW, and the maximum loading point shifts. The movements with the highest iliac bone stress in descending order were: WOF, LF, and NW. In addition, the maximum loading point was located at the sacroiliac joint during NW and WOF, and it shifted to the greater sciatic notch during LF.

## 4. Discussion

This study proceeded from analyzing the differences in ground reaction phases, generating models of joints and muscle strength at the lower extremities, and stress changes of the iliac bones during two progressive motions of Bafa Wubu and NW to demonstrate the biomechanical performance of the Bafa Wubu form of TC. This study also explored the positive effects of Bafa Wubu in enhancing gait motions and effective fitness methods for walking.

### 4.1. Ground Reaction Force Characteristics Analysis

As an essential metric in biomechanics, ground reaction force (GRF) reveals [25] component force exerted on the ground in the sagittal, vertical, and frontal planes during gait. Based on the observed peaks of GRF, it was found that during WOF, GRF was significantly smaller than during NW in the vertical and sagittal axes, whereas GRF during LF was significantly smaller than GRF during NW in the sagittal axis, as a result of the unique gait features of Bafa Wubu. The movement is characterized by the integration of softness and hardness, such that both starting and landing should be gentle. In addition, Bafa Wubu and walking are similar in that the performer’s body moves on the frontal axis and body weight gently shifts from the left leg to the right leg, requiring the performer to keep his body upright and ensure harmony between the upper and lower body. Transitions between empty gaits and solid gaits call for powerful manipulation of the lower extremity joints and muscles, so the force exerted on the ground is supposed to be slow and soft. This is the so-called catwalk boxing mechanism [26]. It is consistent with Zhu’s findings that the ground reaction force of Tai Chi movements is significantly less than that of normal walking, as Zhu showed by comparing four typical Tai Chi movements with normal walking. His research demonstrates that during Tai Chi, a performer’s lower extremity is supposed to be slowly landed, and this calls for power manipulation of the lower extremity joints. Such a gentle and controlled mode of motion is conducive to exercising the lower extremity joints. However, the quantitative analysis of motion speed and direction is limited in Zhu’s research. The present experiment paid attention to analyzing the motion time and the direction of ground reaction force in normal walking and two progressive motions in Bafa Wubu. It was found that the ground reaction of the two progressive motions is significantly smaller that observed during normal walking in the vertical and ant–post direction. Therefore, practitioners are advised to raise the leg and land it slowly over a relatively long period, and slow down the speed of walking through slowly manipulating the lower extremity joints, subsequently enhancing the controlling capacity of the lower extremity. Past studies have shown that improved controlling capacity of the lower extremity can increase the pace length of walking, so that a relatively high gap between the feet can reduce the risk of the heel being caught by obstacles in the swing phase, thus decreasing the likelihood of falling down [27,28].

### 4.2. Lower Extremity Joint Characteristics Analysis

The two progressive motions of Bafa Wubu emphasize that the center of gravity is expected to be low and smooth, and the motions are expected to be gentle and slow in a coherent continuum. Coherence means that stress on the lower extremities is first exerted at the heel. Then, the stress is transmitted to the calf through the ankles and rises to the knee followed by the hip through rotation, finally forming a penetrating force of the three joints of the lower extremities. In this experiment, the vertical stress on three joints is significantly larger than that during NW, indicating that force on the three joints of the lower extremities is penetrating and positively stimulates them. In addition, during WOF and LF, hip joint stress increased significantly in the sagittal axis because Bafa Wubu performers move their lower extremity forward using the strength of the waist and buttocks. It is evident that in Bafa Wubu, motions start from the legs and are manipulated by the waist, and small joints are driven by large joints. Therefore, learners are expected to practice moving their lower extremity on the frontal axis slowly and smoothly using the strength of the hip joints, which can effectively enhance the controlling capacity of the hip joint on the lower extremity and avoid unnecessary energy consumption during walking [29]. Furthermore, people with osteoarthritis or unstable ankles lack the capacity to manipulate their lower extremities in the medial–lateral direction when walking, leading to lateral falls or sprains at the knees and ankles [30]. During WOF and LF, significantly less stress is exerted on the lower extremity joints in the frontal axis compared with that exerted during NW, because stress is undertaken in the vertical and anterior–posterior direction. Therefore, performers are advised to strengthen the manipulation of the lower extremities and reduce stress in the medial–lateral direction as much as possible to improve the controlling capacity of the lower extremities in the medial–lateral direction and reduce the risk of lateral falls during walking.

On the other hand, joint torque is a key element of muscle activities around joints, which plays a pivotal role in movements. The LF of Bafa Wubu is one of the most typical gait trainings in TC, which involves slowly moving the feet forward through the extension and flexion of both knees and ankle joints so that the extension/flexion torque of the knee and ankle is significantly greater than that observed during walking. Hence, regularly practicing progressive motions of Bafa Wubu improves kinaesthesia at the knee joint and ankle joint [31]. Similarly, Cheng and Liang’s [32] experiment verified that 24 weeks of TC training improves the kinesthesia of knee flexion and extension, and 48 weeks of TC training significantly improves the kinesthesia of knee and ankle flexion and extension, because Tai Chi places great emphasis on the movement of the ankle joint. In their research, they found that the muscles, tendons, ligaments, and fascia around the ankle joint are repeatedly stretched; this can improve the flexibility and proprioception of the ankle joint, proving the positive impact of Tai Chi. In addition, although both WOF and LF are forward movements, LF acts as a supplement to the advancing movements of Bafa Wubu, whereby participants’ hip joints are supposed to squat and rotate outwards when they move forward. Therefore, during LF, hip abduction and hip flexion/extension torque were greater than those observed during NW; this result adds to the diversity of hip joint advancements.

### 4.3. Analysis of Lower Extremity Muscle Characteristics

TC is characterized by slow movement and large joint load, involving more human muscles [33]. The two progressive motions described in this paper both start from heel off slowly to toe off, whereby the ankle plantar bone (tibialis anterior) and dorsiflexors (gastrocnemius) are activated more effectively with increasing muscle strength, and muscles around the ankle are obviously stimulated.

Furthermore, when performing WOF and LF, participants displayed greater amounts of muscle strength in the vastus lateralis, vastus medialis, tensor fasciae latae, gluteus maximus, iliopsoas, piriformis, adductor magnus, and gluteus minimus compared with those noted when walking. In Bafa Wubu, gaits have to support eight sides, and this is realized by shifts of body weight between the legs. More specifically, the lower extremity muscles generate force not only forward but also upward, downward, inwards, and outwards symmetrically together with the abduction, adduction, flexion, and extension of the hip joint and knee joint. Additionally, the muscles around the thighs and buttocks repeatedly perform concentric and eccentric contractions. Hence, Bafa Wubu is conducive to improving lower extremity muscle strength and controlling capacity [34].

### 4.4. Lower Extremity Neuromuscular Characteristics Analysis

Based on comparisons of iliac bone stress during two progressive motions and NW, it was found that the stress threshold and the maximum loading point of the iliac bone change with movement. The movements with the greatest iliac bone stress in descending order were: WOF, LF, and NW. In addition, the maximum loading point was located at the sacroiliac joint during NW and WOF, and shifted to the greater sciatic notch during LF. It is noteworthy that when participants perform LF, their hip joints have to squat and rotate outwards. Therefore, the end of the iliac bone is mainly loaded, while primary learners tend to ignore the added strength of external rotation. Previous studies have discovered that changes in the bones are mainly attributed to external forces, so that when bone stress increases, bone trabeculae grow more densely, more osteoblasts are activated, and bone density increases [35]. This finding is consistent with the findings that Bafa Wubu training improves bone density and reduces bone loss because iliac bone stress is enhanced [36]. Furthermore, given that the iliac bone matter supports the human body during walking, Bafa Wubu training can comprehensively stimulate the iliac bone and improve the supporting stability of the lower extremities during NW.

## 5. Conclusions

This is the first study demonstrating the biomechanical performance of Bafa Wubu in professional athletes of Tai Chi. During two progressive motions of Bafa Wubu, the hip, knee, and ankle joints work synergistically to ensure that both feet are gently lifted and landed, resulting in a smaller ground reaction force compared with NW. In addition, load is concentrated on the vertical axes of the lower extremity joints when TC practitioners perform ward off with steps forward and lay with steps forward. Thus, it is recommended to reduce load in the horizontal direction. Furthermore, warding off with steps forward imposes greater effects on the knee and ankle joints, whereas laying with steps forward imposes greater effects on the hip joint. The two progressive motions and NW activate different types of muscles at different levels, and primary learners are expected to emphasize the external rotation of hip joints when performing laying with steps forward. When they practice these two progressive motions and walk, the threshold loading and maximum loading points of their iliac bones are different, varying with the movements. In addition, this study is intended to be developed further to explore specific functions and the practical value of Tai Chi Chuan through long-term intervention.

## Figures and Tables

**Table 1:** Subject Body Parameters (mean ± SD).

Body Parameters	(Mean ± SD)
Age (y)	20.50 ± 1.60
BMI (kg/m^2^)	23.13 ± 1.49
Height (cm)	175.00 ± 5.24
Weight (kg)	70.75 ± 3.77
Leg length (cm)	85.94 ± 2.40
Hip width (cm)	23.13 ± 1.96
Knee width (cm)	9.94 ± 0.68
Ankle width (cm)	7.38 ± 0.52

Due to the design of the AnyBody musculoskeletal model, the model parameters need to be changed according to subjects’ body shapes; therefore, the body shape parameters of the subjects are collected.

**Table 2:** Means and standard deviations (mean ± SD) of the maximum ground reaction force (GRF) during two progressive motions and NW. Units: N/BW.

Parameters	NW	WOF	*p*-Value	LF	*p*-Value
F(ML)	0.58 ± 0.26	0.51 ± 0.10	0.54	0.42 ± 0.12	0.331
F(VT)	10.64 ± 0.66	9.73 ± 0.77 **	0.006	10.13 ± 0.37	0.16
F(AP)	1.89 ± 0.27	1.42 ± 0.21 **	0.002	1.15 ± 0.30 **	0.000

** *p* < 0.01 represents the comparison between two progressive motions and NW. ML: mediolateral, VT: vertical, AP: anteroposterior.

**Table 3:** Mean and standard deviation (mean ± SD) of the maximum joint force and joint torque of the hip, knee and ankle during the two progressive motions and NW.

Joint Force (Units: Nm/BW)		NW	WOF	*p*-Value	LF	*p*-Value
Hip joint	ML	−5.96 ± 2.56	−0.12 ± 0.09 **	0.004	−0.75 ± 0.46 **	0.008
	VT	26.05 ± 10.40	74.62 ± 39.66 **	0.003	76.95 ± 41.47 **	0.002
	AP	1.32 ± 1.03	20.75 ± 14.30 **	0.000	21.28 ± 6.39 **	0.000
Knee joint	ML	−17.16 ± 7.21	−0.56 ± 2.37 **	0.001	−1.15 ± 0.32 **	0.005
	VT	0.22 ± 0.79	3.73 ± 3.11 **	0.009	7.32 ± 2.95 **	0.000
	AP	−0.56 ± 0.42	0.32 ± 0.23	0.54	0.15 ± 0.11	0.423
Ankle joint	ML	−19.40 ± 9.17	−0.11 ± 0.20 **	0.000	−0.20 ± 0.21 **	0.001
	VT	12.41 ± 3.23	18.29 ± 2.06 *	0.042	18.37 ± 4.23 *	0.023
	AP	2.63 ± 0.51	3.13 ± 0.86	0.657	3.54 ± 0.90	0.721
Joint torque (Units: Nm/BW)						
Hip abduction		0.34 ± 0.13	0.41 ± 0.13	0.24	0.53 ± 0.17 *	0.048
Hip external rotation		0.43 ± 0.14	0.34 ± 0.24	0.328	0.20 ± 0.07	0.425
Hip flexion/extension		0.10 ± 0.04	0.18 ± 0.04	0.23	0.36 ± 0.23 **	0.006
Knee flexion/extension		0.23 ± 0.11	0.53 ± 0.16 **	0.004	0.33 ± 0.26	0.21
Ankle flexion/extension		0.07 ± 0.07	0.55 ± 0.34 **	0.008	0.57 ± 0.45 **	0.007

** *p* < 0.01 and * *p* < 0.05 represent comparisons between two progressive motions and NW. ML: mediolateral, VT: vertical, AP: anteroposterior.

**Table 4:** Mean and standard deviation (mean ± SD) of muscle strength during WOF, LF, and NW. Units: N/BW.

Muscles	NW	WOF	*p*-Value	LF	*p*-Value
Vastus lateralis	1.38 ± 1.93	11.70 ± 2.51 **	0.000	23.73 ± 3.35 **	0.000
Vastus medialis	0.68 ± 0.96	7.10 ± 5.37 **	0.002	12.09 ± 4.59 **	0.000
Biceps femoris	4.75 ± 2.01	5.12 ± 2.29	0.35	5.11 ± 2.96	0.441
Semitendinosus	2.23 ± 1.09	2.61 ± 1.20	0.648	2.63 ± 1.86	0.56
Semimembranosus	1.45 ± 0.31	2.61 ± 0.45	0.31	2.37 ± 0.27	0.228
Tensor fasciae latae	0.17 ± 0.21	0.76 ± 0.39 **	0.003	0.57 ± 0.04 **	0.005
Sartorius	0.99 ± 0.61	1.85 ± 0.51	0.46	2.04 ± 0.51	0.698
Gluteus maximus	1.94 ± 1.20	11.50 ± 5.61 **	0.000	13.47 ± 7.32 **	0.000
Gluteus minimus	2.05 ± 0.91	2.89 ± 1.06	0.627	3.84 ± 0.87 **	0.009
Iliopsoas	0.49 ± 0.29	50.71 ± 28.12 **	0.000	57.19 ± 26.19 **	0.000
Piriformis	0.56 ± 0.36	1.43 ± 0.71 **	0.007	1.59 ± 0.62 **	0.002
Adductor longus	0.31 ± 0.27	0.51 ± 0.28	0.421	0.40 ± 0.26	0.64
Adductor magnus	0.37 ± 0.12	7.85 ± 3.50 **	0.006	8.18 ± 3.59 **	0.002
Tibialis anterior	1.55 ± 0.97	4.87 ± 1.30 **	0.001	4.85 ± 1.71 **	0.002
Gastrocnemius	2.72 ± 0.52	6.02 ± 1.94 **	0.000	3.96 ± 1.92	0.562

** *p* < 0.01 represents comparisons between two progressive motions and NW.

## Data Availability

The datasets generated during the present study are available from the corresponding author upon request.

## Abbreviations

TC: Tai Chi/Tai Chi Chuan; BMI: Body mass index; NW: Normal walking; WOF: Warding off with steps forward; LF: Laying with steps forward; GRF: Ground reaction force; ML: mediolateral; VT: vertical; AP: anteroposterior.
